# Two-Year Clinical Outcomes of Transvaginal Radiofrequency Ablation for Symptomatic Uterine Fibroids: A Retrospective Observational Study

**DOI:** 10.3390/jcm15041518

**Published:** 2026-02-14

**Authors:** Mª Eugenia Marín Martínez, Gema Vaquero Argüello, Tirso Pérez Medina, Victoria E. Rey, Mª Luisa de la Cruz Conty, Sara Cruz Melguizo

**Affiliations:** 1 Hospital Infanta Cristina, 28981 Parla, Madrid, Spain; mariaeugenia.marin@salud.madrid.org; 2 Escuela de Doctorado de Medicina y Cirugía, Universidad Autónoma de Madrid, 28029 Madrid, Spain; 3 Hospital Universitario Puerta de Hierro, 28222 Majadahonda, Madrid, Spaintirso.perez@salud.madrid.org (T.P.M.); 4 Clínica Victoria Rey, 41018 Sevilla, Spain; reyvic2026@gmail.com; 5 Departamento de Estadística e Investigación Operativa, Facultad de Medicina, Universidad Complutense de Madrid, 28040 Madrid, Spain; 6 Hospital Universitario San Jorge-Jaca, 22700 Huesca, Spain

**Keywords:** fibroids, transvaginal radiofrequency ablation, heavy menstrual bleeding, minimally invasive techniques, uterine preservation

## Abstract

**Background:** Transvaginal radiofrequency ablation (TRFA) is a minimally invasive, uterus-preserving technique for symptomatic uterine fibroids. This study evaluates its two-year clinical and volumetric outcomes, safety profile, patient satisfaction, and reintervention rates. **Methods:** In this single-center, retrospective, single-arm observational cohort study, 121 premenopausal women underwent outpatient TRFA under general anesthesia between 2018 and 2023. Follow-up visits at 1, 6, 12, and 24 months assessed fibroid volume reduction, symptom improvement using the Uterine Fibroid Symptom and Quality of Life Questionnaire (UFS-QoL), vascularity, satisfaction, complications, and the need for reintervention. A total of 169 fibroids were treated. **Results:** TRFA resulted in progressive fibroid shrinkage, with a mean volume reduction of 57.97% at 6 months and 60.75% at 24 months, accompanied by sustained improvement in UFS-QoL scores (from 30.19 at baseline to 14.97 at 24 months). Patient satisfaction was high (96.61%). Complications were infrequent and predominantly mild, and recovery was rapid, with short postoperative analgesia (mean 2.87 days) and limited sick leave (mean 3.34 days). The two-year reintervention rate was 24.79%, with a substantial proportion corresponding hysteroscopic procedures planned a priori as part of a sequential therapeutic strategy. Among 22 pregnancies recorded after TRFA, 81.82% resulted in term deliveries with favorable neonatal outcomes although fertility was not a predefined study endpoint. **Conclusions:** TRFA appears to be a safe, effective, and well-tolerated minimally invasive treatment for symptomatic uterine fibroids, offering durable symptom relief and significant volume reduction and rapid recovery, and encouraging reproductive outcomes. Within the limitations of its single-arm observational design, these results support TRFA as a valuable uterus-preserving therapeutic option.

## 1. Introduction

Uterine fibroids are benign monoclonal tumors arising from the myometrial muscle and constitute the most common neoplasm of the female reproductive tract, affecting 70–80% of women of reproductive age [1,2].

Their etiology is multifactorial and involves genetic, hormonal, and environmental factors. Although most patients remain asymptomatic, approximately 30% experience symptoms such as menorrhagia, dyspareunia, pelvic pain, pressure-related complaints, or reproductive dysfunction. Clinical severity depends on the size, number, and location of the fibroids, as well as on patient-specific characteristics including age and hormonal status and tends to be more pronounced in women of African descent [3]. Fibroids negatively affect quality of life and impose a substantial burden on healthcare systems [4].

Conventional management of uterine fibroids includes both medical and surgical therapies, each with variable efficacy and limitations. Pharmacological treatments provide symptomatic relief but are often limited by recurrence once therapy is discontinued. Myomectomy allows for uterine preservation; however, postoperative recovery and obstetric implications vary according to the surgical approach. Hysterectomy offers a definitive solution but results in permanent loss of fertility and may carry long-term metabolic consequences even when the ovaries are preserved [5,6,7].

In response to the growing demand for uterus-preserving alternatives, several minimally invasive techniques have been developed, including transvaginal radiofrequency ablation (TRFA).

TRFA involves the delivery of high-frequency alternating electrical current (350–500 kHz) through an ultrasound-guided electrode inserted into the fibroid, generating localized thermal energy (60–100 °C) that induces coagulative necrosis and subsequent fibroid volume reduction.

Beyond immediate thermal injury, TRFA leads to progressive devascularization of the treated fibroid, resulting in ischemia and gradual tissue resorption. These vascular changes, together with the reduction in fibroid volume, contribute to sustained symptom relief over time, particularly with respect to bleeding and pressure-related symptoms.

Since its approval by the U.S. Food and Drug Administration in 2012 for the treatment of uterine fibroids, TRFA has evolved through different approaches. The laparoscopic (Acessa) and transcervical (Sonata) techniques were initially introduced, followed more recently by the transvaginal approach, which allows ultrasound-guided needle insertion through the vaginal wall without abdominal incisions or cervical dilation. Overall, TRFA aims to reduce fibroid volume and improve associated symptoms [8,9,10,11].

The aim of this study is to evaluate the two-year clinical outcomes of transvaginal radiofrequency ablation for symptomatic uterine fibroids, focusing on therapeutic efficacy, safety, and the need for reintervention.

## 2. Materials and Methods

**Figure 1:**
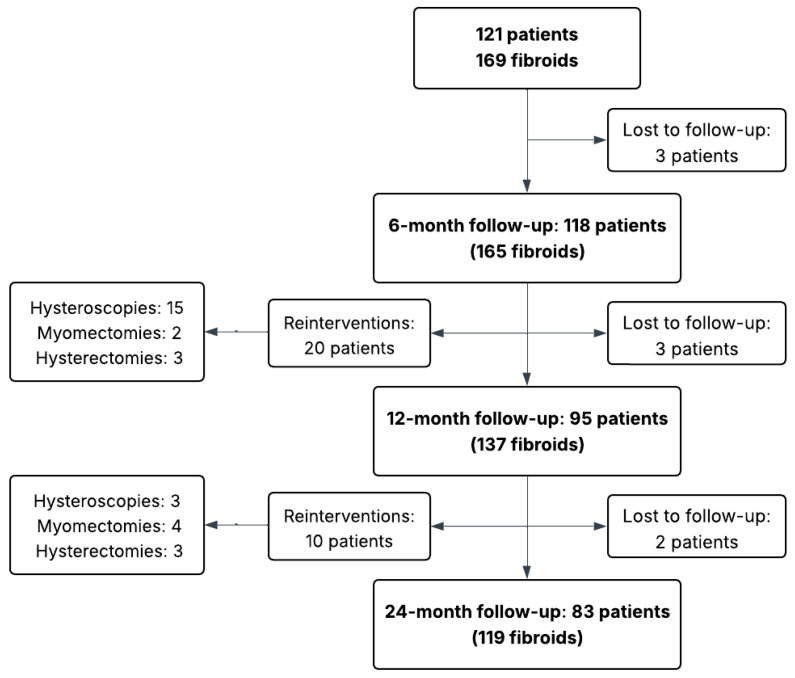
Flow diagram of the study.

This was a single-center, retrospective, single-arm observational cohort study, including a 24-month follow-up of 121 patients who underwent TRFA for symptomatic uterine fibroids at Hospital Puerta de Hierro (Madrid, Spain) between July 2018 and November 2023 (Figure 1). The primary outcomes were therapeutic efficacy, safety, patient satisfaction, and the need for reintervention. Written informed consent for the anonymized use of clinical data and images was obtained from all participants at the time of the intervention. The retrospective analysis of these data was approved by the Institutional Review Board.

**Inclusion and Exclusion Criteria:** Eligible patients were women aged ≥ 18 years with symptomatic fibroids, defined as menstrual bleeding requiring sanitary product changes more often than every 2 h for at least one day, and with a fibroid volume ≤ 220 cm^3^ or multiple fibroids totaling ≤ 220 cm^3^. Exclusion criteria included fibroids > 220 cm^3^, FIGO type 7 lesions or those without vaginal access, current pregnancy, active pelvic inflammatory disease, or pelvic infection within the preceding 3 months. Ongoing hormonal therapy or other symptomatic treatments were permitted.

**Preoperative Assessment:** Before the procedure, all patients underwent a comprehensive gynecological examination and two-dimensional Doppler ultrasound. Fibroids were classified according to the FIGO system [12,13], and vascularity was assessed following the MUSA criteria [14]. The three largest orthogonal diameters were recorded for each fibroid, and volume was calculated using the formula 0.5233 × a × b × c. Symptom burden and quality of life were evaluated using the Uterine Fibroid Symptom and Health-Related Quality of Life Questionnaire (UFS-QoL) [15,16].

In eight patients with symptomatic fibroids containing a submucosal component and a Lasmar-STEPW score ≥5 [17], TRFA was used as an adjunctive treatment to reduce fibroid volume and vascularization prior to a planned hysteroscopic resection.

**Figure 2:**
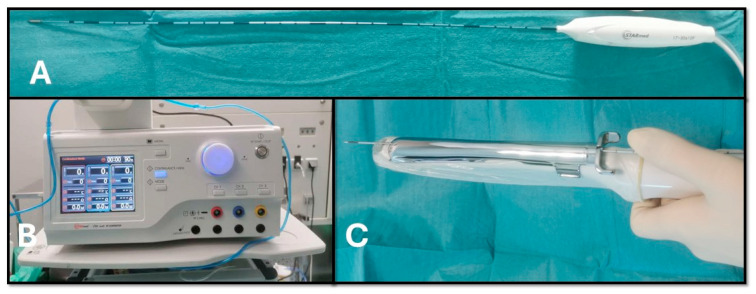
(**A**) RFA electrode. (**B**) TRFA generator. (**C**) Vaginal transducer with guide. Image created by the author.

**TRFA Procedure:** Procedures were performed under general anesthesia. Surgery was scheduled independently of the menstrual cycle and required no specific preparation. Prophylactic intravenous cefazolin (2 g) and dexamethasone (8 mg) were administered. Patients were positioned in lithotomy. Two dispersive electrodes were placed on the inner thighs, and the radiofrequency generator was connected to a cold saline bag to allow continuous electrode cooling. TRFA was performed using the STARmed generator (JJP Hospitalaria S.L., Sevilla, Spain) at a fixed power of 100 W. A 35 cm, 17-gauge needle electrode with a 10 mm active tip (REF 17-35s10F) was used under real-time guidance with a Philips Affiniti 70W ultrasound system, assisted by a 15-gauge metal guide placed on the vaginal probe (Figure 2). Until January 2021, an ultrasound-guided biopsy was performed before ablation using an 18-gauge, 30 cm semiautomatic needle (M-Biopsy REF N 30118030, Stenløse, Denmarkø). Biopsy was subsequently discontinued due to its limited diagnostic yield and the adequacy of imaging criteria to confirm benignity, as TRFA is contraindicated in fibroids that do not meet benign imaging features. Thereafter, fibroid benignity was assessed exclusively using strict sonographic criteria. Imaging features considered compatible with benign fibroids included well-defined margins and regular contours, homogeneous echogenicity, absence of central necrosis or irregular cystic areas, lack of rapid growth on serial follow-up, predominantly peripheral or low vascularity without aberrant central vessels (according to MUSA criteria), and a stable relationship with the surrounding myometrium, with no signs of infiltration. Fibroids showing imaging features suspicious for malignancy were excluded from TRFA.

**Figure 3:**
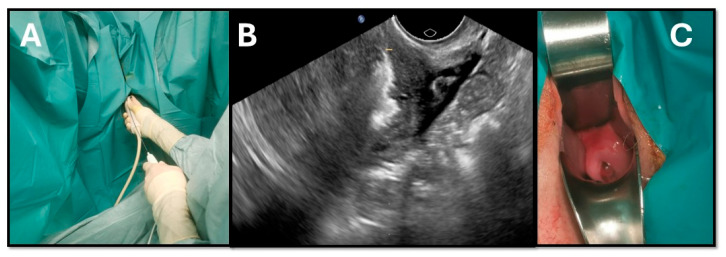
(**A**) Transducer with radiofrequency electrode. (**B**) Ultrasound appearance of ablation within the fibroid. (**C**) Transvaginal puncture sites after TRFA. Image created by the author.

The goal of TRFA was to induce 1 cm^3^ necrotic areas within the fibroid, with an average ablation time of approximately 10 s per application. Treatment was considered complete when ablation lines covered all ultrasound sections of the fibroid (Figure 3).

The procedure was carried out on an outpatient basis. Postoperative analgesia consisted of mild oral agents as needed. Antibiotics or corticosteroids were not prescribed. Patients were advised to avoid high-impact exercise for one week.

**Figure 4:**
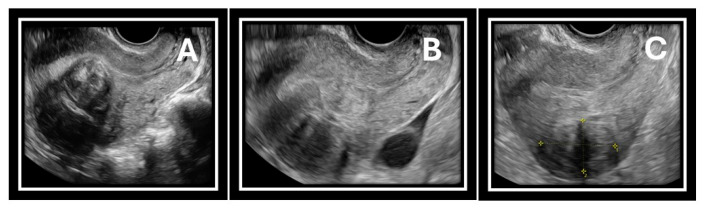
(**A**) Fibroid before TRFA. (**B**) Fibroid 6 months after TRFA. (**C**) Fibroid 12 months after TRFA.

**Follow-Up:** Follow-up visits were scheduled at 1, 6, 12, and 24 months. The first postoperative visit assessed symptom evolution, patient satisfaction, analgesic use, and duration of sick leave. Unscheduled medical visits and complications were documented and classified according to the Clavien–Dindo system [18]. At 6 and 24 months, patients completed the UFS-QoL questionnaire again, and Doppler ultrasound assessed fibroid size and vascularity (Figure 4). The need for additional medical therapy for bleeding control or for reintervention was documented.

Statistical analysis: Descriptive statistical analysis was performed using SPSS version 29 (IBM Corp., Armonk, NY, USA). Variables analyzed included baseline patient and fibroid characteristics, symptom evolution (pre- and post-TRFA UFS-QoL), procedural details, reintervention type, and subsequent reproductive outcomes. Categorical variables were summarized as absolute and relative frequencies (percentages), while continuous variables were described using the mean or the median, and the range.

Longitudinal changes in fibroid volume and UFS-QoL symptom severity scores were analyzed using linear mixed-effects models to account for repeated measurements and clustering within patients. Time since treatment was included as a fixed effect, and a random intercept for patient was specified in all models. Fibroid volume models were adjusted for baseline fibroid size (<50 vs. ≥50 cm^3^), fibroid type (grouped into clinically meaningful categories), fibroid location, number of fibroids, concomitant medical therapy during follow-up, and planned sequential hysteroscopy. UFS-QoL symptom severity scores were analyzed at the patient level, with models adjusted for age, parity, number of fibroids, concomitant medical therapy during follow-up (yes/no), and planned sequential hysteroscopy (yes/no). Fibroids classified as FIGO type 8 were described descriptively but excluded from predictor analyses due to their atypical location and low frequency. Model estimates are reported with 95% confidence intervals. Sensitivity analyses excluding patients undergoing planned sequential hysteroscopy were performed to assess the robustness of the findings. These analyses were conducted using R 4.5.0 (R Core Team, Vienna, Austria). AI tools (ChatGPT 5.2) were used solely for language editing; scientific content is fully author-generated.

## 3. Results

Figure 1 presents the flow diagram of the study. A total of 121 premenopausal patients were treated, with 169 fibroids ablated (mean 1.4 fibroids per patient). The mean age of the cohort was 40.85 years. Baseline demographic, clinical and fibroid characteristics are summarized in Table 1.

The most common fibroid location was posterior (42.01%) and anterior (40.24%), with fundal localization being less frequent. According to the FIGO classification, type 3 and combined type 2–5 fibroids were the most prevalent. Mean fibroid volume was 32.25 cm^3^, with a mean maximum diameter of 3.75 cm. Vascularity assessed by MUSA criteria was type 2 in 98.82% of cases. The mean baseline UFS-QoL symptom severity score was 30.19. All TRFA procedures were completed on an outpatient basis. Mean ablation time per fibroid was 238 s. Patient satisfaction was high, with 96.61% reporting being satisfied or very satisfied. Recovery was rapid, with short sick leave duration and limited need for postoperative analgesia. Procedure-related characteristics are detailed in Table 2.

Complications were minimal: grade I occurred in 4.13% (mild vaginal bleeding), grade II in 1.65% (inflammatory syndrome treated with corticosteroids), grade IIIa in 12.39% (office hysteroscopy), and grade IIIb in 3.30% (including one bowel perforation and three operative hysteroscopies). The patient with bowel perforation presented 48 h after the procedure with progressive abdominal pain and required exploratory laparoscopy with segmental bowel resection and primary anastomosis, with complete postoperative recovery. No grade IV or V complications were observed.

Reintervention was required in 24.79% of patients, at a median of 8 months after TRFA. Most reinterventions consisted of hysteroscopic procedures, 44.4% of which had been preplanned as part of a complementary sequential treatment strategy following TRFA, while myomectomy and hysterectomy were less common. The rate of unplanned reintervention was 18.18% (Table 3).

**Fibroid Volume Reduction, Vascularity, and Symptom Improvement**: Progressive reduction in fibroid volume was observed throughout follow-up, accompanied by symptomatic improvement. At 6 months, mean fibroid volume reduction exceeded 50%. Although most fibroids decreased in size, a small proportion increased in volume (7/165; 4.24%). At this point, 87.88% exhibited MUSA type 1 vascularity, and the mean UFS-QoL score had improved to 16.88. These findings remained stable at 12 months and further improved at 24 months, with increasing rates of MUSA type 1 vascularity and sustained symptom relief (Table 4).

**Need for Additional Treatment at 24 Months:** Among patients evaluated at 24 months (*n* = 83), 68.67% required no further treatment. When complementary medical management was needed, it mainly consisted of hormonal therapy, with tranexamic acid used in a minority of cases (Table 4).

**Longitudinal analysis of fibroid volume and symptom severity:**

Linear mixed-effects modeling demonstrated a significant and progressive reduction in fibroid volume over time following TRFA. Compared with baseline, mean fibroid volume decreased significantly at all follow-up visits, with the largest reduction observed at 24 months, supporting a sustained treatment effect rather than a transient volume change (Supplementary Table S1). Baseline fibroid size was the strongest independent determinant of absolute volume throughout follow-up, with larger fibroids remaining larger in absolute terms despite treatment-related volume reduction. Fibroid type was associated with differences in absolute volume after adjustment, whereas fibroid location was not independently associated with volume. A higher number of fibroids was associated with smaller mean fibroid volume, consistent with a distribution of total fibroid burden across multiple lesions. Concomitant medical therapy during follow-up was not independently associated with fibroid volume. Planned sequential hysteroscopy showed a non-significant trend toward lower volume; however, sensitivity analyses excluding these patients yielded consistent results, confirming the robustness of the observed volume reduction over time (Supplementary Table S2).

In parallel, UFS-QoL symptom severity scores improved markedly and significantly over time. Compared with baseline, UFS-QoL scores decreased substantially at 6 months and continued to improve at later follow-up visits, indicating a sustained symptomatic benefit following TRFA (Supplementary Table S3). Time since treatment was the strongest determinant of symptom improvement, while age, parity, and number of fibroids were not independently associated with symptom severity. Concomitant medical therapy during follow-up was independently associated with higher UFS-QoL scores, suggesting greater residual symptom burden among patients requiring additional medical treatment. Planned sequential hysteroscopy was not independently associated with symptom severity, and sensitivity analyses excluding these patients confirmed that the observed improvement in symptoms was not driven by sequential surgical management (Supplementary Table S4).

**Reproductive Outcomes**: A total of 22 pregnancies occurred after TRFA. Fertility was not a predefined study endpoint, but pregnancies had been recorded as a relevant clinical outcome. Of these, 81.82% resulted in term deliveries, with a cesarean section rate of 27.78%. Obstetric complications were uncommon and limited to postpartum hemorrhage. Median time from TRFA to conception was 11.5 months. Perinatal outcomes are summarized in Table 5.

## 4. Discussion

The findings of this study support the efficacy and safety of TRFA as a minimally invasive therapeutic alternative for symptomatic uterine fibroids. The most pronounced clinical effect was observed during the first six months after treatment, reflecting an early and meaningful reduction in symptoms. These initial benefits remained stable at 12 and 24 months, suggesting sustained clinical efficacy over time. Importantly, the results of the mixed-effects models suggest that volumetric reduction following TRFA is accompanied by sustained symptomatic improvement. Our results align with previously published studies, reporting comparable trends in symptom improvement and volumetric reduction following TRFA [8,9,11,19].

Smaller fibroids demonstrated a greater volume reduction and more pronounced symptomatic improvement. Among the 169 fibroids treated, 113 achieved a reduction greater than 50% at six months, most of which corresponded to myomas with lower baseline volume (mean: 23.26 cm^3^; range: 0.3–138 cm^3^). This response pattern reinforces the influence of baseline fibroid size on treatment efficacy, consistent with the findings of Santalla-Hernández et al., who identified initial volume as a key prognostic factor in radiofrequency ablation [9].

The primary methodological limitation of this study is its single-arm observational design and the absence of a comparison group, which restricts the ability to draw direct comparisons with established therapeutic alternatives such as myomectomy, uterine artery embolization (UAE), or medical management. The heterogeneity of clinical indications and patient preferences did not allow the establishment of a homogeneous comparative group without introducing selection bias; therefore, a non-randomized observational design was adopted. In addition, the single-center nature of the study may limit the generalizability of the results to other settings with different levels of expertise or technological availability. Furthermore, the presence of planned sequential treatments, such as complementary hysteroscopic procedures in selected cases, may introduce additional bias in the interpretation of clinical outcomes and reintervention rates. These limitations should be considered when interpreting the findings and support the need for future multicenter comparative studies to further validate these findings.

Patient selection criteria for TRFA are still evolving, as this is a relatively recent and increasingly adopted technique. An absolute limitation remains the requirement for adequate vaginal access to the fibroid, regardless of its size or FIGO classification. In addition, available evidence suggests that in cases of large fibroids or multiple lesions, the risk of adverse effects related to extensive coagulative necrosis may be increased. These considerations highlight the importance of careful patient selection and the need to further refine indication criteria as clinical experience with TRFA continues to accumulate.

The decision to discontinue routine pre-ablation biopsy was based on its limited diagnostic yield and the use of strict imaging-based selection criteria. This approach was applied cautiously, excluding cases with features suspicious for malignancy or considered at higher risk, and should be interpreted within this context.

TRFA provides symptomatic improvement comparable to myomectomy, with the advantages of ambulatory performance and shorter recovery time. However, its utility may be limited in cases of multiple or large fibroids, in which surgical resection remains more appropriate [20,21]. Compared with UAE, TRFA is associated with less postoperative pain, reduced analgesic requirements, and a faster return to normal activities. UAE, although offering sustained symptom control, carries a reported reintervention rate of 29–33% at five years [22,23].

Oral GnRH antagonists have demonstrated substantial efficacy in reducing menstrual bleeding and improving quality of life, but their impact on fibroid volume is modest. Long-term use requires add-back therapy, temporarily restricts fertility, and is associated with symptom recurrence after discontinuation [24,25].

In this study, the TRFA reintervention rate (24.79%) was comparable to that of myomectomy series. Importantly, a substantial proportion (44.4%) of hysteroscopic procedures were intentionally planned as part of a sequential approach, particularly in submucosal fibroids with high Lasmar-STEPW scores—reflecting a deliberate therapeutic strategy rather than failure of the initial procedure [26].

Although rare, serious complications may occur. In our series, one case of bowel perforation required surgical management and resulted in complete recovery, underscoring the importance of appropriate training and standardized procedural safeguards.

Regarding reproductive outcomes, available evidence on fertility following TRFA remains limited but encouraging. Reported pregnancy and live birth rates appear comparable to those achieved with other minimally invasive fibroid treatments, although current data are insufficient to draw definitive conclusions [27,28]. In our cohort, although reproductive outcomes after TRFA were also favorable, fertility assessment was not a primary objective of the study but rather an additional clinical outcome observed during follow-up.

In line with the ongoing shift toward increasingly less invasive approaches in gynecology, TRFA can be regarded as a therapeutic option that aligns with recent literature highlighting the growing role of ultra-minimally invasive surgery and its favorable clinical outcomes [29,30].

## 5. Conclusions

Transvaginal radiofrequency ablation appeared to be an effective, safe, and well-tolerated treatment for symptomatic uterine fibroids, with a high patient satisfaction rate (96.61%) and an ambulatory profile that enables rapid recovery. Over the 24-month follow-up period, a progressive reduction in fibroid volume was observed (up to 60.75%), together with sustained improvement in quality-of-life scores and significant symptom relief. Although reinterventions occurred in 24.79% of patients, most consisted of hysteroscopic procedures intentionally planned as part of a sequential therapeutic strategy, facilitated by TRFA to optimize myoma resection. Complications were infrequent and generally mild, supporting the favorable safety profile of the technique. Reproductive outcomes were encouraging, with 81.82% of pregnancies resulting in term deliveries and favorable neonatal parameters. Given the observational nature of the study, these results reflect observed associations rather than causal relationships and should be interpreted in light of the single-arm design and the absence of a comparison group.

While larger studies are needed to confirm these findings, TRFA emerges as a strong therapeutic option for women with symptomatic fibroids, particularly those seeking uterine preservation and a faster return to daily activities. Within this context, TRFA represents a minimally invasive approach that aligns with current trends toward uterus-preserving and patient-centered gynecologic care.

## Figures and Tables

**Table 1:** Patient and treated fibroid characteristics.

Patients (*n* = 121)	Age: mean (range)	40.85 years old (25–56)
	Ethnicity:	
	Caucasian	73 (60.33%)
	Latin American	42 (34.71%)
	African American	2 (1.65%)
	Asian	1 (0.83%)
	North African	3 (2.48%)
	Parity:	
	Nulliparous	61 (50.41%)
	Multiparous	60 (49.59%)
	Main symptoms:	
	Heavy menstrual bleeding	70 (57.85%)
	Heavy menstrual bleeding and compressive	43 (35.54%)
	Heavy menstrual bleeding and infertility	8 (6.61%)
	Treatment prior to TRFA	121 (100%)
Fibroids (*n* = 169)	Number per patient: mean (range)	1.40 (1–5)
	Location:	
	Anterior	68 (40.24%)
	Posterior	71 (42.01%)
	Fundus	27 (15.98%)
	Cervical	3 (1.77%)
	FIGO fibroid type:	
	Type 1	2 (1.18%)
	Type 2	10 (5.92%)
	Type 3	35 (20.71%)
	Type 4	27 (15.98%)
	Type 5	20 (11.83%)
	Type 6	6 (3.55%)
	Type 8	3 (1.78%)
	Type 2–5	47 (27.81%)
	Type 2–4	12 (7.10%)
	Type 3–5	7 (4.14%)
	Volume: mean (range)	32.25 cm^3^ (0.3–218.84)
	Maximum diameter: mean (range)	3.75 cm (0.8–9)
	MUSA vascularization:	
	Type 2	167 (98.82%)
	Type 3	2 (1.18%)
	UFS-QoL prior to TRFA: mean (range)	30.19 (12–40)

Data are expressed as frequencies (percentages), unless otherwise indicated.

**Table 2:** Procedure characteristics.

Ablation Time Per Fibroid: Mean (Range)	238 s (10–840)
Patient satisfaction with the technique:	
Very satisfied	60/118 (50.85%)
Satisfied	54/118 (45.76%)
Somewhat dissatisfied	4/118 (3.39%)
Dissatisfied	0/118 (0%)
Sick leave: mean (range)	3.34 days (0–15)
Postoperative analgesia: mean (range)	2.87 days (0–8)
Procedure on an outpatient basis	121/121 (100%)
Complications classified according to Clavien–Dindo:	
Grade I: mild vaginal bleeding	5/121 (4.13%)
Grade II: inflammatory syndrome	2/121 (1.65%)
Grade III:	
3a: Hysteroscopy for myoma resection in consultation	15/121 (12.39%)
3b:	
Intestinal perforation	1/121 (0.83%)
Surgical hysteroscopy	3/121 ^a^ (2.48%)
Grade IV	0/121 (0.0%)
Grade V	0/121 (0.0%)

Data are expressed as frequencies (percentages), unless otherwise indicated. ^a^ All 3 at 7 months after TRFA.

**Table 3:** Reinterventions.

Frequency of reinterventions	30/121 (24.79%)
Time to reintervention: median (range)	8 months (7–22) ^a^
Type of reintervention:	
Hysteroscopy:	18/30 (60.0%) ^a^
Operating room	3/18 ^b^
Consultation room	15/18 ^b^
Myomectomy	6/30 (20.0%)
Hysterectomy	6/30 (20.0%)
Rate of unplanned reintervention	22/121 (18.18%)

Data are expressed as frequencies (percentages), unless otherwise indicated. ^a^ 8/18 (44.4%) hysteroscopic procedures had been preplanned as complementary treatment. ^b^ Complete resection in 100% of cases.

**Table 4:** Post-procedural outcomes in fibroid volume and symptoms.

At 6 months	Percentage reduction in fibroid volume: mean (range)	57.97% (from 112.22% increase to 100% reduction)
	Maximum fibroid diameter: mean (range)	2.68 cm (0–8.4)
	MUSA vascularization:	
	Type 1	145/165(87.88%)
	Type 2	19/165 (11.51%)
	Type 3	1/165 (0.61%)
	UFS-Qo	16.88 (8–34)
At 12 months	Percentage reduction in fibroid volume: mean (range)	58.17% (from 434% increase ^a^ to 100% reduction)
	Maximum fibroid diameter: mean (range)	2.47 cm (0–8.4)
	MUSA vascularization:	
	Type 1	117/137 (85.40%)
	Type 2	20/137 (14.60%)
At 24 months	Percentage reduction in fibroid volume: mean (range)	60.75% (from 340.56% increase to 100% reduction)
	Maximum fibroid diameter: mean (range)	2.27 cm (0 to 8.9 cm)
	MUSA vascularization:	
	Type 1	108/119 (90.76%)
	Type 2	11/119 (9.24%)
	UFS-Qo	14.97 (8–28)
	Complementary treatment:	
	No treatment	57/83 (68.67%)
	Tranexamic acid	5/83 (6.03%)
	Progestogens (oral or IUD)	21/83 (25.30%)

Data are expressed as frequencies (percentages), unless otherwise indicated. ^a^ In pregnant patient.

**Table 5:** Pregnancies after TRFA.

Number of pregnancies	22
Types:	
First trimester abortions	4 (18.18%)
Full-term pregnancies:	18 (81.82%)
Cesarean sections	5/18 (27.78%)
Eutocic delivery	9/18 (50.0%)
Instrumental delivery	4/18 (22.22%)
Complications	2 ^a^ (11.11%)
Time from TRFA to pregnancy: median (range)	11.5 months (3–23)
Gestational age at birth: median (range)	40 weeks (37 + 6 to 41 + 4)
Birth weight: median (range)	3.400 kg (2.660–3.880)

Data are expressed as frequencies (percentages), unless otherwise indicated. ^a^ 2 postpartum hemorrhage.

## Data Availability

Clinical data supporting the findings of this study are not publicly available as they contain sensitive patient information and no public repository has been created for this dataset. Data may be provided in anonymized form by the corresponding author upon reasonable request and subject to ethical approval.

## Supplementary Materials

The following supporting information can be downloaded at: https://www.mdpi.com/article/10.3390/jcm15041518/s1.

## References

[1] Laughlin S.K., Schroeder J.C., Baird D.D. (2010). New directions in the epidemiology of uterine fibroids. Semin. Reprod. Med..

[2] Wise L.A., Laughlin-Tommaso S.K. (2016). Epidemiology of Uterine Fibroids: From Menarche to Menopause. Clin. Obstet. Gynecol..

[3] Stewart E.A., Laughlin-Tommaso S.K., Catherino W.H., Lalitkumar S., Gupta D., Vollenhoven B. (2016). Uterine fibroids. Nat. Rev. Dis. Primers.

[4] Cardozo E.R., Clark A.D., Banks N.K., Henne M.B., Stegmann B.J., Segars J.H. (2012). The estimated annual cost of uterine leiomyomata in the United States. Am. J. Obstet. Gynecol..

[5] Donnez J., Dolmans M.M. (2016). Uterine fibroid management: From the present to the future. Hum. Reprod. Update.

[6] Chen Y., Li F., Liang L., Hua H., Liu S., Yu Z., Chen Q., Huang S., Qin P. (2024). Examining the association of hysterectomy with and without oophorectomy on cardiovascular disease and all-cause, cardiovascular or cancer mortality: A systematic review and meta-analysis. BJOG Int. J. Obstet. Gynaecol..

[7] Wang Z., Li X., Zhang D. (2022). Impact of hysterectomy on cardiovascular disease and different subtypes: A meta-analysis. Arch. Gynecol. Obstet..

[8] Bradley L.D., Pasic R.P., Miller L.E. (2019). Clinical Performance of Radiofrequency Ablation for Treatment of Uterine Fibroids: Systematic Review and Meta-Analysis of Prospective Studies. J. Laparoendosc. Adv. Surg. Tech. A.

[9] Santalla-Hernández Á., Naveiro-Fuentes M., Benito-Villena R., López-Criado M.S., González-Paredes A., Fernández-Parra J. (2022). Efficacy, Complications, and Factors Predictive of Response to Treatment with Transvaginal Radiofrequency Ablation for Symptomatic Uterine Myomas. J. Minim. Invasive Gynecol..

[10] Iversen H., Dueholm M. (2017). Radiofrequency Thermal Ablation for Uterine Myomas: Long-term Clinical Outcomes and Reinterventions. J. Minim. Invasive Gynecol..

[11] Rey V.E., Labrador R., Falcon M., Garcia-Benitez J.L. (2019). Transvaginal Radiofrequency Ablation of Myomas: Technique, Outcomes, and Complications. J. Laparoendosc. Adv. Surg. Tech. A.

[12] Munro M.G., Critchley H.O., Fraser I.S., FIGO Menstrual Disorders Committee (2018). The two FIGO systems for normal and abnormal uterine bleeding symptoms and classification of causes of abnormal uterine bleeding in the reproductive years: 2018 revisions. Int. J. Gynaecol. Obstet..

[13] Munro M.G., Critchley H.O.D., Fraser I.S. (2011). The flexible FIGO classification concept for underlying causes of abnormal uterine bleeding. Semin. Reprod. Med..

[14] Van den Bosch T., Dueholm M., Leone F.P.G., Valentin L., Rasmussen C.K., Votino A., Van Schoubroeck D., Landolfo C., Installé A.J.F., Guerriero S. (2015). Terms, definitions and measurements to describe sonographic features of myometrium and uterine masses: A consensus opinion from the Morphological Uterus Sonographic Assessment (MUSA) group. Ultrasound Obstet. Gynecol..

[15] Spies J.B., Coyne K., Guaou Guaou N., Boyle D., Skyrnarz-Murphy K., Gonzalves S.M. (2002). The UFS-QOL, a new disease-specific symptom and health-related quality of life questionnaire for leiomyomata. Obstet. Gynecol..

[16] Calaf J., Palacios S., Cristóbal I., Cañete M.L., Monleón J., Fernández J., Hernández A., Vázquez F. (2020). Validation of the Spanish version of the Uterine Fibroid Symptom and Quality of Life (UFS-QoL) questionnaire in women with uterine myomatosis. Med. Clin..

[17] Lasmar R.B., Barrozo P.R.M., Dias R., de Oliveira M.A.P. (2005). Submucous myomas: A new presurgical classification to evaluate the viability of hysteroscopic surgical treatment--preliminary report. J. Minim. Invasive Gynecol..

[18] Clavien P.A., Barkun J., de Oliveira M.L., Vauthey J.N., Dindo D., Schulick R.D., de Santibañes E., Pekolj J., Slankamenac K., Bassi C. (2009). The Clavien-Dindo classification of surgical complications: Five-year experience. Ann. Surg..

[19] Chen I., Berman J.M., Balk E.M., Saldanha I.J., Kowalczewski E., Yi J., Zanotti S., Al Hilli M., Kho K.A. (2025). Radiofrequency Ablation for the Treatment of Uterine Fibroids: A Systematic Review and Meta-Analysis by the AAGL Practice Committee. J. Minim. Invasive Gynecol..

[20] Fasciani A., Turtulici G., Pedullà A., Sirito R. (2023). Uterine Myoma Position-based Radiofrequency Ablation (UMP-b RFA): 36 months follow-up clinical outcomes. Eur. J. Obstet. Gynecol. Reprod. Biol..

[21] Don E.E., Mijatovic V., van Eekelen R., Hehenkamp W.J.K., Huirne J.A.F. (2023). The Effect of a Myomectomy on Myoma-related Symptoms and Quality of Life: A Retrospective Cohort Study. J. Minim. Invasive Gynecol..

[22] de Bruijn A.M., Ankum W.M., Reekers J.A., Birnie E., van der Kooij S.M., Volkers N.A., Hehenkamp W.J. (2016). Uterine artery embolization vs. hysterectomy in the treatment of symptomatic uterine fibroids: 10-year outcomes from the randomized EMMY trial. Am. J. Obstet. Gynecol..

[23] Stewart E.A., Laughlin-Tommaso S.K. (2024). Uterine Fibroids. N. Engl. J. Med..

[24] Donnez J., Becker C.M., Mangler M., Paszkowski M., Paszkowski T., St-Pierre J., Ionescu-Ittu R., Boolell M., Bestel E., Hori S. (2025). Linzagolix rapidly reduces heavy menstrual bleeding in women with uterine fibroids: An analysis of the PRIMROSE 1 and 2 trials. Fertil. Steril..

[25] Syed Y.Y. (2022). Relugolix/Estradiol/Norethisterone (Norethindrone) Acetate: A Review in Symptomatic Uterine Fibroids. Drugs.

[26] Marín Martínez M.E., Vaquero Argüello G., Pérez Medina T., Calles Sastre L.B., De la Cruz Conty M.L., Cruz Melguizo S. (2025). Combined use of Radiofrequency ablation and hysteroscopy in the treatment of uterine Myomas: An innovative approach. Eur. J. Obstet. Gynecol. Reprod. Biol..

[27] Marín Martínez M.E., Cruz-Melguizo S., Vaquero Argüello G., Engels Calvo V., De la Cruz Conty M.L., Pérez Medina T. (2024). Transvaginal radiofrequency ablation: A therapeutic option for managing symptomatic uterine fibroids in women with reproductive desires. F S Rep..

[28] Rey V.E., Falcon M.M., Ferrara I., Yanes G. (2025). Pregnancy Outcomes After Transvaginal Radiofrequency Ablation of Leiomyomas. Obstet. Gynecol..

[29] La Verde M., Riemma G., Tropea A., Biondi A., Cianci S. (2022). Ultra-minimally invasive surgery in gynecological patients: A review of the literature. Updates Surg..

[30] Morris J.M., Liang A., Fleckenstein K., Singh B., Segars J. (2023). A Systematic Review of Minimally Invasive Approaches to Uterine Fibroid Treatment for Improving Quality of Life and Fibroid-Associated Symptoms. Reprod. Sci..

