# Supplementary material for: Two-Year Clinical Outcomes of Transvaginal Radiofrequency Ablation for Symptomatic Uterine Fibroids: A Retrospective Observational Study
Source: J Clin Med. 2026 Feb 14;15(4):1518. doi: 10.3390/jcm15041518 (PMC12942411; doi:10.3390/jcm15041518)
Supplement: Supplementary file 1 [file jcm-15-01518-s001.zip › jcm-4084430-supplementary.pdf]

Supplementary Table S1. Linear mixed-effects model results for fibroid volume.

	Estimate	CI_low	CI_high	p_value
(Intercept)	33.02665	24.86136	41.63602	0.00000
TIEMPO2	-6.61836	-9.78051	-3.42248	0.00004
TIEMPO3	-7.00427	-10.37554	-3.60689	0.00005
TIEMPO4	-8.15263	-11.66844	-4.61772	0.00001
TAMANO_BASAL1	60.18665	53.94283	66.51913	0.00000
LOCALIZACION2	-0.82763	-4.98471	3.29423	0.69089
LOCALIZACION3	3.18025	-2.15368	8.51874	0.24235
TIPO_CAT2	-8.15842	-12.89652	-3.50252	0.00055
TIPO_CAT3	-6.82060	-11.92231	-1.71277	0.00792
N_MIOMAS	-4.76517	-8.73887	-0.86588	0.01868
Terapia_concomitante1	-2.36302	-8.92306	4.12652	0.47418
Planned_sequential_hysteroscopy1	-8.46483	-17.59842	0.47686	0.06548

Variable definitions:

Time since TRFA (TIEMPO):
1 = before TRFA;
2 = 6 months after TRFA;
3 = 1 year after TRFA;
4 = 2 years after TRFA.

Baseline fibroid size (TAMANO_BASAL):
0 = <50 cm³;
1 = ≥ 50 cm³.

Fibroid location (LOCALIZACION):
1 = Anterior;
2 = Posterior;
3 = Fundal.

Fibroid type (TIPO_CAT):
1 = Submucosal;
2 = Intramural;
3 = Subserosal.

Number of fibroids (N_MIOMAS): Number of fibroids per patient.

Concomitant medical therapy during follow-up (Terapia_concomitante):
Hormonal therapy or Amchafibrin.
0 = No;
1 = Yes.

Planned sequential hysteroscopy (Planned_sequential_hysteroscopy):
0 = No;
1 = Yes.

Supplementary Table S2. Sensitivity analysis of the linear mixed-effects model for fibroid volume excluding patients undergoing planned sequential hysteroscopy.

	Estimate	CI_low	CI_high	p_value
(Intercept)	33.21027	24.51253	42.40467	0.00000
TIEMPO2	-6.50635	-9.89511	-3.08296	0.00017
TIEMPO3	-6.81109	-10.30646	-3.28847	0.00014
TIEMPO4	-8.00019	-11.62368	-4.35794	0.00002
TAMANO_BASAL1	59.97695	53.40482	66.64614	0.00000
LOCALIZACION2	-0.90474	-5.24768	3.40861	0.67771
LOCALIZACION3	3.89172	-1.72421	9.51291	0.17428
TIPO_CAT2	-7.25126	-12.34082	-2.22488	0.00474
TIPO_CAT3	-6.24420	-11.50809	-0.97847	0.01895
N_MIOMAS	-5.29289	-9.58784	-1.11036	0.01542
Terapia_concomitante1	-2.22463	-9.14852	4.62961	0.52284

Supplementary Table S3. Linear mixed-effects model results for UFS-QoL scores.

	Estimate	CI_low	CI_high	p_value
(Intercept)	27.48902	22.89781	32.08908	0.00000
TIEMPO2	-13.27068	-14.29564	-12.24792	0.00000
TIEMPO3	-14.86995	-16.03291	-13.71360	0.00000
edad	0.05928	-0.06309	0.18148	0.34003
PARIDAD1	0.34557	-1.19003	1.87570	0.65673
N_MIOMAS	-0.64942	-1.59649	0.29809	0.17808
Terapia_concomitante1	2.77517	1.24120	4.31188	0.00051
Planned_sequential_hysteroscopy1	1.25411	-0.88762	3.39141	0.24934

Variable definitions:

Time since TRFA (TIEMPO):
1 = before TRFA;
2 = 6 months after TRFA;
3 = 2 years after TRFA.

Patient age (edad): Age in years at the time of TRFA.

Parity (PARIDAD): History of childbirth.
0 = No previous deliveries (nulliparous);
1 = One or more previous deliveries (parous).

Number of fibroids (N_MIOMAS): Number of fibroids per patient.

Concomitant medical therapy during follow-up (Terapia_concomitante):
Hormonal therapy or Amchafibrin.
0 = No;
1 = Yes.

Planned sequential hysteroscopy (Planned_sequential_hysteroscopy):
0 = No;
1 = Yes.

Supplementary Table S4. Sensitivity analysis of the linear mixed-effects model for UFS-QoL scores excluding patients undergoing planned sequential hysteroscopy.

	Estimate	CI_low	CI_high	p_value
(Intercept)	27.91963	23.00352	32.85282	0.00000
TIEMPO2	-13.12549	-14.22875	-12.02498	0.00000
TIEMPO3	-14.76439	-15.95460	-13.58280	0.00000
edad	0.05375	-0.07921	0.18633	0.42499
PARIDAD1	0.15775	-1.53395	1.84247	0.85364
N_MIOMAS	-0.78853	-1.77924	0.20214	0.11839
Terapia_concomitante1	2.80794	1.24187	4.37812	0.00059
